# Forkhead Box Protein P3 (FOXP3) Represses ATF3 Transcriptional Activity

**DOI:** 10.3390/ijms222111400

**Published:** 2021-10-22

**Authors:** Chiung-Min Wang, William Harry Yang, Leticia Cardoso, Ninoska Gutierrez, Richard Henry Yang, Wei-Hsiung Yang

**Affiliations:** Department of Biomedical Sciences, Mercer University School of Medicine, Savannah, GA 31404, USA; chiungminw@gmail.com (C.-M.W.); theyangbossman@gmail.com (W.H.Y.); leticiacdn@gmail.com (L.C.); NinMGutierrez@stu.southuniversity.edu (N.G.); thelegoman700@gmail.com (R.H.Y.)

**Keywords:** FOXP3, ATF3, post-translational modification, transcriptional activity

## Abstract

Activating transcription factor 3 (ATF3), a transcription factor and acute stress sensor, is rapidly induced by a variety of pathophysiological signals and is essential in the complex processes in cellular stress response. FOXP3, a well-known breast and prostate tumor suppressor from the X chromosome, is a novel transcriptional repressor for several oncogenes. However, it remains unknown whether ATF3 is the target protein of FOXP3. Herein, we demonstrate that ATF3 expression is regulated by FOXP3. Firstly, we observed that overexpression of FOXP3 reduced ATF3 protein level. Moreover, knockdown FOXP3 by siRNA increased ATF3 expression. Secondly, FOXP3 dose-dependently reduced ATF3 promoter activity in the luciferase reporter assay. Since FOXP3 is regulated by post-translational modifications (PTMs), we next investigated whether PTMs affect FOXP3-mediated ATF3 expression. Interestingly, we observed that phosphorylation mutation on FOXP3 (Y342F) significantly abolished FOXP3-mediated ATF3 expression. However, other PTM mutations on FOXP3, including S418 phosphorylation, K263 acetylation and ubiquitination, and K268 acetylation and ubiquitination, did not alter FOXP3-mediated ATF3 expression. Finally, the FOXP3 binding site was found on ATF3 promoter region by deletion and mutagenesis analysis. Taken together, our results suggest that FOXP3 functions as a novel regulator of ATF3 and that this novel event may be involved in tumor development and progression.

## 1. Introduction

Transcription factor Forkhead Box Protein P3 (FOXP3), encoded from the X chromosome (Xp11.23 in human), was originally identified as the causative mutation for lethal X-linked autoimmune/poly-endocrine dysregulation syndrome [1,2,3,4]. Unlike other FOX proteins, FOXP3 is mainly expressed in a subset of CD4+ T cells which function as suppressors in the immune system. In the T cell lineage, FOXP3 is essential for regulatory T (Treg) cell development and maintenance of immune homeostasis, as evidence shows that mutations of FOXP3 results in defective development of CD4+ CD25+ Treg cells [5]. Therefore, FOXP3 has become the most specific biomarker of Treg cells in the immunosuppressive system and homeostasis. Animal model studies have concluded that deficient and/or truncated FOXP3 is lethal due to Treg cell deficiency [5,6]. In humans, many mutations and/or alternatively spliced variants of the FOXP3 gene are strongly associated with an extremely rare and severe autoimmune disorder termed immunodysregulation, poly-endocrinopathy and enteropathy, X-linked syndrome (IPEX) [7]. Currently, the only curative treatment for IPEX is an allogeneic stem cell transplant from a healthy donor.

In addition to CD4+ T cells, FOXP3 also expresses in a variety of normal tissues, such as breast, liver, lung, prostate, spleen, pituitary, testis, and thymus [8,9,10,11], suggesting that FOXP3 might have broad biological and physiological functions. Extensive studies in the past 20 years have strongly suggested that FOXP3 is a novel tumor suppressor, especially in breast, colon, and prostate cancers. For example, deletions and mutations of FOXP3 have been found in human breast and prostate cancer samples, and germline mutations of FOXP3 result in a high rate of spontaneous breast cancer and fatal autoimmunity in mice [12]. Secondly, as a tumor suppressor, FOXP3 represses many key target genes in cancer development and progression, such as HER2/ERBB2 [12], BRCA1 [13], SKP2 [14], and CD44 [15], providing a strong link between FOXP3 and cell cycle regulation as well as FOXP3 and DNA repair system. Thirdly, the FOXP3-miR-146-NF-κB as an oncotarget and its axis has a functional role during tumor initiation in both prostate and breast cancers [16,17]. Moreover, miR-155, induced by FOXP3 through transcriptional repression of BRCA1, is associated with tumor initiation in human breast cancer, suggesting that plasma miR-155 may serve as a non-invasive biomarker for detection of early-stage breast cancer [18]. Furthermore, miR-141 and miR-200c are regulated by a FOXP3-KAT2B axis and are associated with tumor metastasis in breast cancer, suggesting that circulating levels of miR-141 and miR-200c are also potential biomarkers for early detection of breast cancer metastases [19]. Finally, loss of FOXP3 and TSC1 accelerates prostate cancer progression through synergistic regulation of c-MYC [20]. However, FOXP3 has been shown to promote cancer growth and metastasis in non-small cell lung cancer [21,22]. Recently, tumor CD274 (PD-L1) expression is inversely associated with FOXP3+ cell density in colorectal cancer tissues [23] and FOXP3 is expressed significantly higher in cytolytic-high colorectal tumors [24]. Together, these results demonstrate that FOXP3 has broad functions as a tumor suppressor in breast and prostate cancers and a tumor promoter in non-small cell lung cancer, suggesting that the regulatory machinery associating with FOXP3 in each cancer type might be critical for FOXP3 function.

Activating transcription factor 3 (ATF3), which is a member of the basic leucine zipper family of transcription factors, acts through binding to the ATF/cAMP response element (CRE) found in a number of promoters of key regulatory proteins that determine cell fate, circadian signaling, and homeostasis [25]. ATF3, as an immediate gene, is rapidly induced in cells once exposed to stress stimuli, including those initiated by cytokines, genotoxic agents, infections, reactive oxygen species, nerve injury, tissue damage, inflammation, and/or essential physiological stresses [25,26]. In addition, some evidence has implicated that ATF3 is up-regulated in many cancers, suggesting that ATF3 is an oncogene [27,28]. However, other evidence has indicated that ATF3 is able to suppress cell proliferation and inhibit the development of tumors [29,30,31]. Moreover, hepatocyte ATF3 is a key regulator of high-density lipoprotein and bile acid metabolism in the development of atherosclerosis [32]. Importantly, our group is the first to demonstrate that SUMOylation of ATF3 alters its transcriptional activity on regulation of TP53 gene [33], and that loss of SUMOylation on ATF3 inhibits proliferation of prostate cancer cells by modulating CCND1/2 activity [34]. These results imply that ATF3 has broad biological and physiological functions including cancer development and metabolism.

Previously, our group demonstrated that FOXP3 directly regulates UBC9 (the only E2 enzyme for SUMOylation) expression, suggesting that FOXP3 has a potential effect on regulating the global protein SUMOylation process [35]. Though FOXP3 has an essential and critical role in autoimmunity, cancer development, and Treg development, with hundreds of FOXP3 target genes already identified in both cancer cells and Treg cells, the functional role of FOXP3 in regulating ATF3 is largely unknown. Therefore, since FOXP3 is a transcription factor and has broad biological and physiological effects in cells and organs, we assessed the function of FOXP3 in ATF3 transcriptional activity using several human cell lines in the present study.

## 2. Results

### 2.1. FOXP3 Decreases ATF3 Protein Level

**Figure 1:**
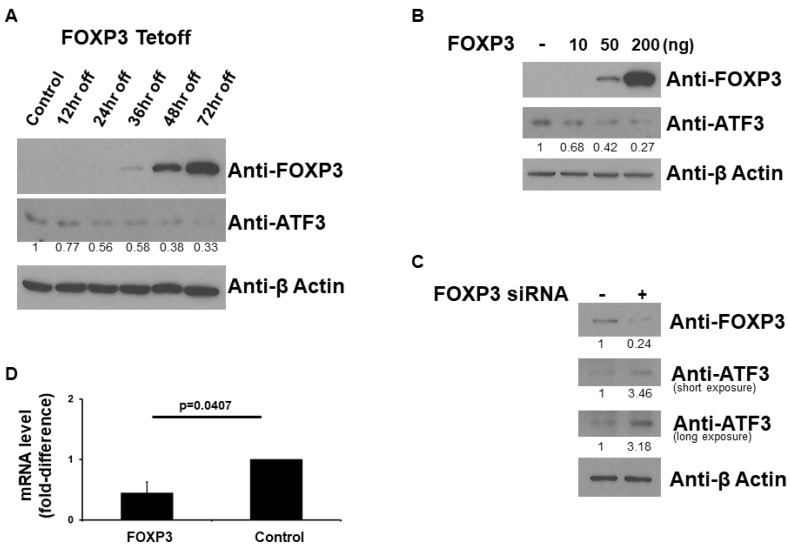
FOXP3 decreases ATF3 protein level. (**A**) Western blot analysis of ATF3 expression from FOXP3-Tet-off MCF7 cells. (**B**) Western blot analysis of ATF3 expression from FOXP3 over-expressed MCF7 cells. (**C**) Western blot analysis of ATF3 expression from HEK293 cells treated with FOXP3 siRNA. The expression levels of FOXP3 and ATF3 were determined using anti-FOXP3 and anti-ATF3 immunoblotting, respectively. The β-Actin levels were also determined for equal loading. (**D**) Real-time RT-PCR analysis of ATF3 expression by FOXP3 from MCF7 human breast cancer cells. Total RNA was extracted from cells and then reverse transcribed to cDNA followed by qPCR analysis with glyceraldehyde-3-phosphate dehydrogenase (GAPDH) as an internal control. The experiments were performed two times, each with triplicate samples. Error bars indicate standard errors.

Since FOXP3 is a transcription factor, we first investigated the role of FOXP3 on ATF3 expression. We used FOXP3-Tet-off MCF7 cells to evaluate whether FOXP3 affects ATF3 protein expression since MCF7 (human breast cancer) cells express very small amounts of FOXP3. As shown in Figure 1A, FOXP3 induction by doxycycline removal decreased the expression levels of ATF3. We next over-expressed FOXP3 in MCF7 cells to evaluate whether FOXP3 affects ATF3 protein expression. As shown in Figure 1B, FOXP3 dose-dependently decreased the expression levels of ATF3. In order to confirm the results of Figure 1A,B, we next knockdown FOXP3 by siRNA system in HEK293 (human embryonic kidney) cells, which express more endogenous FOXP3 than MCF7 cells, to evaluate whether FOXP3 affects ATF3 protein expression. As shown in Figure 1C, reduction of FOXP3 by siRNA increased the expression levels of ATF3. In order to confirm the previous findings, we next performed qRT-PCR experiments. The expression vectors encoding wild-type FOXP3 or empty vectors were transfected into MCF7 human breast cancer cells. As shown in Figure 1D, when wild-type FOXP3 was transfected, the level of ATF3 mRNA was significantly decreased (approximately 50% reduction). Overall, these findings indicate that FOXP3 has the potential to down-regulate ATF3 expression.

### 2.2. FOXP3 Is a Repressor of the ATF3 Promoter

**Figure 2:**
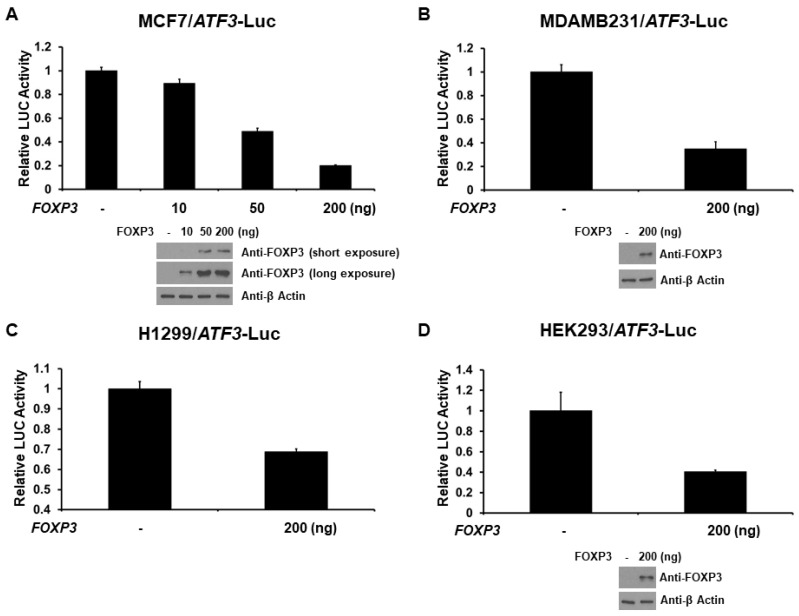
FOXP3 represses ATF3 transcription. (**A**) MCF7, (**B**) MDAMB231, (**C**) H1299, and (**D**) HEK293 cells were co-transfected, where indicated, with different amount of FOXP3 expression plasmid and ATF3 promoter-LUC reporter plasmid. Luciferase activities were measured 48 h after transfection and normalized with *Renilla* activity. Relative LUC activity was calculated and plotted. The protein levels of FOXP3 and β Actin in the cells from the reporter assays were confirmed using anti-FOXP3 and anti-β Actin immunoblotting, respectively.

As FOXP3 decreases ATF3 protein expression, we next investigated the role of FOXP3 on ATF3 promoter activation. The −1372 bp ATF3 promoter-LUC reporter plasmid was co-transfected with FOXP3 expression plasmid into several different cell lines (MCF7, MDAMB231, H1299, or HEK293 cells) and ATF3 promoter activity was determined by measuring the LUC activity in cell lysates 48 h after transfection. As shown in Figure 2A, expression of FOXP3 generated a dose-dependent decrease in the activity of ATF3 gene transcription in MCF7 cells. Similar results were observed in MDAMB231 (triple negative human breast cancer), H1299 (human lung cancer), and HEK293 cells. This finding indicates that FOXP3 is a repressor of ATF3 transcription independent on cell types.

### 2.3. Minimal ATF3 Promoter Region Responsive to FOXP3 Repression

To determine whether the FOXP3 response elements (REs) are required for FOXP3-mediated ATF3 expression, we first searched for potential FOXP3 binding site(s) on the ATF3 promoter region using the ALGGEN-PROMO website (http://alggen.lsi.upc.es/cgi-bin/promo_v3/promo/promoinit.cgi?dirDB=TF_8.3, access on: 1 August 2021) and rVista 2.0 website (https://rvista.dcode.org, access on: 1 August 2021). We identified one potential FOXP3 binding site on the human ATF3 promoter region. The potential FOXP3 binding site is located 870 bp (AAAAAAAAATCGAACCGATAC) upstream of the transcription start site, suggesting that FOXP3 may regulate ATF3 transcription directly.

**Figure 3:**
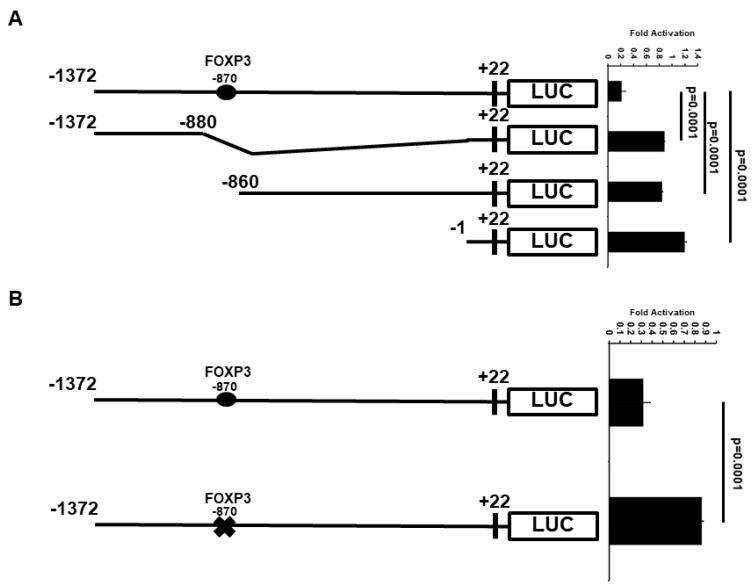

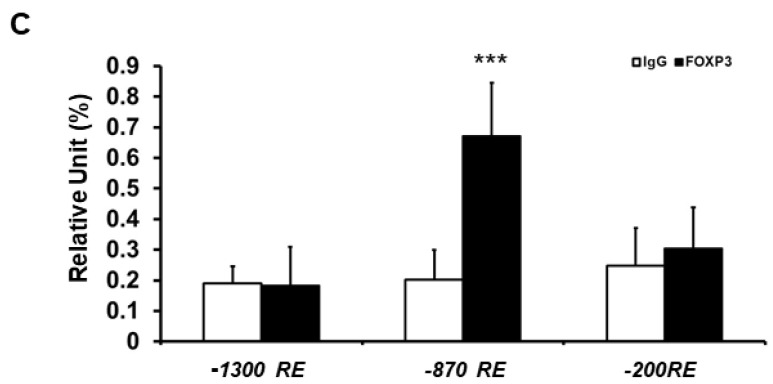
Regions of ATF3 promoter important for transcriptional down-regulation by FOXP3. (**A**) MCF7 cells were co-transfected with ATF3 promoter deletion constructs and FOXP3 expression plasmids. Luciferase activities were measured 48 h after transfection and normalized with *Renilla* activity. Relative LUC activity was calculated and plotted. (**B**) MCF7 cells were co-transfected with FOXP3 expression plasmids and with either wild-type (with −870 FOXP3 RE) or mutant (with −870 FOXP3 RE mutated) ATF3 promoter constructs. Luciferase activities were measured 48 h after transfection and normalized with *Renilla* activity. Relative LUC activity was calculated and plotted. (**C**) The quantification of the amount of DNA fragment precipitated (expressed as relative unit as a percentage of the total input DNA) in chromotin immunoprecipitation (ChIP) assay and qPCR analysis of possible FOXP3-binding sites (−200 RE, −870 RE, and −1300 RE) in the ATF3 promoter region in MCF7 cells. The experiments were performed two times. *** indicates *p* < 0.001 vs. IgG.

Because the −1372 bp ATF3 promoter contains one potential candidate of FOXP3 RE, the ATF3 promoter was truncated to determine whether FOXP3 RE is important for transcriptional repression of ATF3 by FOXP3 (Figure 3A). Deletion of the FOXP3 RE (−870 bp) resulted in a major loss of FOXP3-mediated ATF3 transcriptional repression.

We next generated −870 bp mutant (AAAATCGAA→AAAAAAAAA) ATF3 promoter-LUC reporter plasmids. As shown in Figure 3B, mutation of the −870 bp FOXP3 RE resulted in approximately 75% loss of FOXP3-mediated ATF3 promoter repression. Together, these results indicate that the −870 bp RE is essential for the FOXP3 action on the ATF3 promoter. To confirm FOXP3 directly binds to the human ATF3 promoter region, we next performed FOXP3 or immunoglobin (IgG) chromotin immunoprecipitation (ChIP) assay with qPCR analysis. As shown in Figure 3C, FOXP3 strongly binds to the −870 RE region of the ATF3 promoter but not the −200 RE and −1300 RE regions. These results indicate that the −870 RE is the major FOXP3-binding site on the human ATF3 promoter region.

### 2.4. Phosphorylation at Tyr342 of FOXP3 Is Required for Full FOXP3-Mediated ATF3 Transcriptional Activity

**Figure 4:**
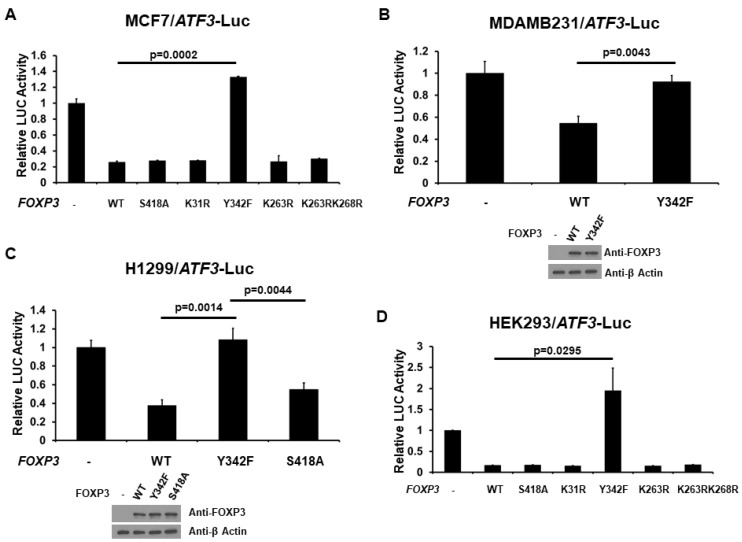
Post-translational modifications of FOXP3 regulate the repression of the ATF3 promoter. (**A**) MCF7, (**B**) MDAMB231, (**C**) H1299, and (**D**) HEK293 cells were co-transfected with the ATF3 promoter-LUC reporter plasmid and either wild-type (WT), S418A, K31R, Y342F, K263R, or K263RK268R FOXP3 expression plasmid. Luciferase activities were measured 48 h after transfection and normalized with *Renilla* activity. Relative LUC activity was calculated and plotted. The protein levels of FOXP3 and β Actin in the cells from the reporter assays were confirmed using anti-FOXP3 and anti-β Actin immunoblotting, respectively.

Because the function of FOXP3 has been shown to be affected by post-translational modifications such as phosphorylation and acetylation [20,35], we next examined the effect of the post-translational modifications of FOXP3 on its transcriptional activity of the ATF3 promoter. MCF7 cells were co-transfected with the ATF3 promoter-LUC reporter plasmid and with either wild-type (WT), K31R (mimicking de-acetylated at K31), K263R (mimicking de-acetylated at K263), K263RK268R (mimicking de-acetylated at both K263 and K268), S418A (mimicking de-phosphorylated at S418), or Y342F (mimicking de-phosphorylated at Y342) FOXP3 expression plasmid. As shown in Figure 4A, in MCF7 cells, while the WT FOXP3 repressed ATF3 promoter activity as expected, K31R, K263R, K263RK268R, and S418A FOXP3 also reduced this effect (similar to WT level). Interestingly, loss of phosphorylation on Y342 abolished FOXP3-mediated ATF3 promoter repression, suggesting that phosphorylation at Y342 is extremely important for FOXP3 on ATF3 promoter activity. Similar results were observed in MDAMB231 and H1299 cells, suggesting that phosphorylation at Y342 is critical for FOXP3 function. Overall, these results suggest that phosphorylation is essential for FOXP3-mediated ATF3 promoter activity.

## 3. Discussion

Within eukaryotic cells, transcription factors (representing the connection of multiple signaling pathways for the growth and development of tissue and organisms) regulate downstream target genes by responding to a wide diversity of physiological and pathological stimuli, and therefore play essential roles in apoptosis, cell growth, developmental control, metabolism, pathogenesis, reproduction, and response to intercellular signals and environment insults [36,37,38]. Deregulation of transcription factors results in many human diseases, such as autoimmune dysfunctions, cardiovascular diseases, metabolic disorders, platelet disorders, and cancers [39,40]. FOXP3, a member of the transcription factor family of FOX proteins, has main functions in autoimmune homeostasis and cancer development and prevention. Herein, we show for the first time that FOXP3 acts as a transcriptional repressor of the human ATF3 gene in human cells.

It is well documented that FOXP3, as a critical master transcription regulator for Treg cell development and function, helps control the activities of various genes (such as oncogenes SKP2 and HER-2/ErbB2) related to the cancer development of the breast and prostate [12,14]. A previous report has shown that FOXP3 maintains Treg unresponsiveness by selectively inhibiting the promoter binding activity of c-Jun-based AP-1 [41]. The structure of AP-1 consists of heterodimers of families of c-Fos, c-Jun, ATF, and JDP. Interestingly, in the present work, we showed that FOXP3 down-regulates ATF3 protein level and decreases ATF3 promoter activity. Our promoter analysis and ChIP assays further supports that FOXP3 response element −870 bp on the ATF3 promoter region is critical for regulating ATF3 gene expression. Our data highlights that ATF3 is the novel target gene for FOXP3.

FOXP3 can cooperate with several transcription factors, and among them is tumor suppressor p53. A previous report has demonstrated that FOXP3 is a key downstream regulator of p53 that is sufficient to induce p21 expression, ROS production and p53-mediated senescence [42]. Many reports, including ours, have demonstrated that ATF3 regulates the stability of p53 and the expression of the TP53 gene [33,43]. The evidence from our group has also highlighted that SUMOylation plays an important role for ATF3-mediated TP53 gene expression [33]. Interestingly, a study has demonstrated that the human ATF3 gene is one of the target genes directly activated by p53 [44], suggesting a functional link between stress-inducible transcriptional repressor ATF3 and p53. However, whether FOXP3 directly involves in p53-ATF3 interaction in a feedback loop remains unknown. Further future studies are indeed necessary to dissect this potential mechanism and regulatory loop.

Post-translational modifications such as acetylation, phosphorylation, methylation, and SUMOylation influence a wide range of cellular activities, including metabolism and cancer development [45,46]. Accumulating evidence indicates that human FOXP3 can be modified by phosphorylation (S418 and Y342), acetylation (K31, K263, and K268), ubiquitylation (K263, K268, and K31), and methylation (R48, R51) [47]. In the present work, we demonstrated that replacement of Y342 by a phenylalanine (F) residue in FOXP3 leads to significant loss of the repression of ATF3’s transcriptional activity. This result is consistent with our previous report that phosphorylation at Y342 on FOXP3 is essential for UBC9 expression [35]. Both reports further highlight the crucial role on FOXP3 as a transcription factor. A previous study has shown that phosphorylation at Y342 of FOXP3 by lymphocyte-specific protein tyrosine kinase (LCK) represses cell invasion [48], suggesting that phosphorylation at Y342 of FOXP3 by LCK plays an important role for ATF3 expression. Interestingly, our current results suggest that another major phosphorylation site (S418) on FOXP3 does not play an important role in ATF3 repression. Since Y342 is modified by LCK and S418 is modified by PP1 from the previous reports, this suggests that LCK-FOXP3 pathway may be a major regulatory pathway for ATF3 regulation. Future studies are indeed necessary to dissect this potential regulatory mechanism.

The activatory/repressing role of splicing events on the neoplastic development/progression has been studied recently in different neoplasms, including alternative splicing of FOXP3. For example, immune responses may be manipulated by modulating the expression of FOXP3 isoforms, which has broad implications for the treatment of autoimmune diseases [49]. Moreover, alternative splicing of FOXP3 controls regulatory T cell effector functions and is associated with human atherosclerotic plaque stability [50]. Accumulated data also suggest that IPEX syndrome may be a consequence of alternatively spliced FOXP3 [51]. Finally, FOXP3 isoform profile has been linked to cardiovascular diseases [52]. Interestingly, SRSF1 has been shown to regulate alternative splicing events, especially in breast cancer [53] and lung cancer [54]. SRSF1 has also been found to be overexpressed in brain glioblastoma [55,56] and to be potentially used as a diagnostic marker of gliomas. With our current results of FOXP3-mediated ATF3 regulation, future studies are indeed necessary to explore the FOXP3 spliced isoforms in ATF3 regulation as well as the role of SRSF1 in FOXP3 isoform regulation, especially in the cancer field.

In conclusion, this study demonstrates that FOXP3, through FOX protein response element, is a novel repressor of ATF3 promoter, and that phosphorylation at Y342 plays a critical role for FOXP3′s transcriptional activity.

## 4. Materials and Methods

### 4.1. Chemicals and Reagents

Both cell culture reagents and cell culture medium were purchased from Thermo Fisher Scientific (Waltham, MA, USA). Antibodies against ATF3, FOXP3, and β-Actin (Santa Cruz Biotechnology Inc., Santa Cruz, CA, USA). Luciferase activity was measured using the Dual-Luciferase Reporter Assay System (Promega, Madison, WI, USA).

### 4.2. DNA Constructs

Human FOXP3-pcDNA6 expression plasmid (with myc and HIS tags) was described previously [35]. S418A, Y342F, K31R, K263R, K263RK268R FOXP3 expression plasmids were created by PCR-based mutagenesis from WT FOXP3-pcDNA6 expression plasmid (QuikChange Lightning site-directed mutagenesis kit, Agilent/Strategene, La Jolla, CA, USA). S418A and Y342F represent the loss of phosphorylation of FOXP3. K31R, K263R, and K263RK268R represent the loss of acetylation of FOXP3. All these mutations have been confirmed by previous studies, including ours. The human ATF3 promoter (−1372/+22 bp) pGL2 plasmid was kindly provided by Dr. Aronheim (Technion-Israel Institute of Technology, Haifa, Israel). The human ATF3 promoter deletion constructs were then generated by removal of specific fragments of DNA sequence in Yang lab. The human ATF3 promoter with FOXP3 RE mutant (−870 bp) plasmids were created by PCR-based mutagenesis (QuikChange Lightning site-directed mutagenesis kit, Agilent/Strategene, La Jolla, CA, USA). All DNA plasmid constructs were verified by Sanger nucleotide sequencing.

### 4.3. Cell Culture and Transfection

H1299, HEK293, MCF7, and MDAMB231 cells were purchased from the American Type Culture Collection (Manassas, VA, USA). The cells were maintained in Dulbecco’s Modified Eagle Medium (DMEM) in the presence of 10% fetal bovine serum and Pen/Strep antibiotics (GIBCO/Life Technologies, Grand Island, NY, USA) in a humidified incubator (5% CO_2_ at 37 °C) and cultured for less than six months. We also routinely checked for Mycoplasma contamination using a PCR-based kit (Millipore-Sigma, Burlington, MA, USA). We chose MCF7 cells for overexpression study and HEK293 cells for siRNA experiments because MCF7 cells have the lowest FOXP3 expression and HEK293 cells have the highest FOXP3 expression among cells we tested. After incubation, the cells were transfected with specific expression plasmids described in each assay using Fugene HD Transfection Reagent (Roche, Madison, WI, USA). Forty-eight hours after transfection, the cells were harvested and lysed and ready for promoter luciferase reporter assays or Western blot analysis.

### 4.4. ATF3 Promoter Luciferase Reporter Assays

Cells were cultured in 24-well plates overnight and then transiently transfected with ATF3 promoter-firefly luciferase plasmid and internal control pRL-TK plasmid (which encodes *Renilla* luciferase activity) in the presence of Fugene HD Transfection Reagent (Roche, Madison, WI, USA). At 48 h after transfection, the cells were harvested and lysed in passive lysis buffer (Promega, Madison, WI, USA). Luminescence was detected with the Dual-Luciferase Reporter Assay System (Promega, Madison, WI, USA) using a luminometer (Turner Designs, Sunnyvale, CA, USA) according to the manufacturer’s instructions. For each reporter assay, we also had a group of three wells without any plasmid transfected to make sure the background signals picked up by a luminometer were low. The firefly luciferase activity was normalized by calculating the ratio to *Renilla* luciferase activity. The relative luciferase activity was calculated as a fold change to the control groups. All experiments were performed three times in a triplicate setting.

### 4.5. Western Blot Analysis

Cells were lysed with RIPA buffer supplemented with protease and phosphatase, and protein contents of the high-speed supernatant were determined using the BCA^TM^ Protein Assay kit assay (Pierced/Thermo Scientific, Rockford, IL, USA). Equivalent quantities of protein (approximately 40 µg) were resolved on 10% polyacrylamide-SDS gels and transferred to polyvinylidene difluoride (PVDF) membrane (Bio-Rad, Hercules, CA, USA) by wet electrophoretic transfer. The membranes were probed with specific primary first and then with specific secondary antibodies. Blots were visualized using the Supersignal West Dura Extended Duration Substrate kit (Pierce Chemical Co., Rockford, IL, USA). The intensity of the protein band was quantified by ImageJ program.

### 4.6. RT-PCR and Real-Time ChIP

Total RNA from MCF7 cells was extracted using the TRIzol reagent (Thermo Fisher Scientific, Waltham, MA, USA) and treated with DNase (Ambion, Austin, TX, USA) to remove genomic DNA. The RNA concentration was quantified by ultraviolet spectrometry before being reverse-transcribed to cDNA. One microgram of total RNA was converted into cDNA using the iScript cDNA synthesis kit (Bio-Rad, Hercules, CA, USA) according to the manufacturer’s instructions. The final cDNA product was purified and eluted in Tris-EDTA buffer using QIAquick PCR purification kits (QIAGEN, Germantown, MD, USA) according to the manufacturer’s instructions. Quantitative PCR (using 5 ng cDNA per μL) was performed on an ABI 7500 qPCR system (Applied Biosystems, Foster City, CA, USA) using TaqMan Universal PCR Master Mix Kit (Applied Biosystems, Foster City, CA, USA) according to the manufacturer’s instructions. Two primers (5′- CCC TTT GTC AAG TGC CG -3′ and 5′- GGCA CTT TGC AGC TGC -3′) were used to amplify 241-bp human ATF3 fragments. Two primers (5′- CAT CAC CAT CTT CCA GGA GCG AG -3′ and 5′- GTC TTC TGG GTG GCA GTG ATG G -3′) were used to amplify 341-bp human glyceraldehydes-3-phosphate dehydrogenase (GAPDH) fragments. For real-time ChIP assays, the extracted DNA fragments were quantified by real-time qPCR using pairs of primers that covered the FOXP3 response region within the human ATF3 promoter. The primers used for −1300 RE PCR were: CAAGAAGGTTCC (forward) and CCTTAAAAACG (reverse). The primers used for −870 RE PCR were: CTTGTCAATTTC (forward) and CTCCGGGCTCC (reverse). The primers used for −200 RE PCR were: GGAACACGCAG (forward) and CTGAGACACACAC (reverse).

### 4.7. Statistical Analysis

Statistical comparisons were performed by using the Student’s *t*-test to determine the statistical significance between groups. A *p* < 0.05 was considered statistically significant between groups.

## 5. Conclusions

In summary, we have shown a novel relationship between FOXP3 and ATF3 for the first time. Our studies suggest FOXP3 is a novel repressor of the ATF3 promoter, and the FOXP3-mediated ATF3 transcription is critically regulated by the phosphorylation at Y342. Overall, our findings add a new layer of information to the previous understanding of FOXP3 functions.

## Data Availability

Data is contained within the article or Supplementary Material.

## Supplementary Materials

The following are available online at https://www.mdpi.com/article/10.3390/ijms222111400/s1.

## Abbreviations

FOXP3	Forkhead box protein P3
ATF3	Activating transcription factor 3
SUMO	Small ubiquitin-like modifier
