# Supplementary figures and images for: Forkhead Box Protein P3 (FOXP3) Represses ATF3 Transcriptional Activity
Source: Int J Mol Sci. 2021 Oct 22;22(21):11400. doi: 10.3390/ijms222111400 (PMC8583784; doi:10.3390/ijms222111400)

Foxp3 tetoff and overexpression data

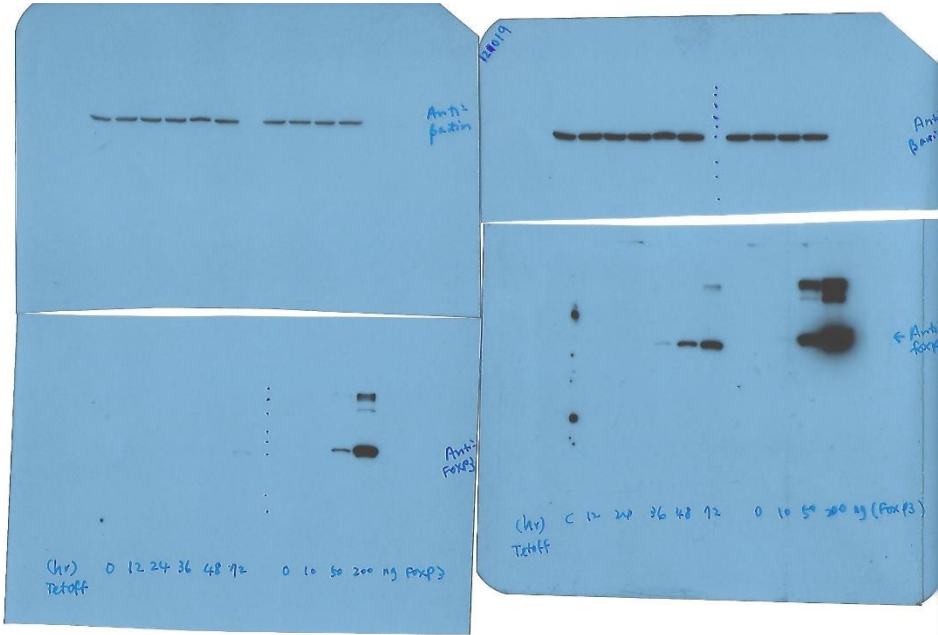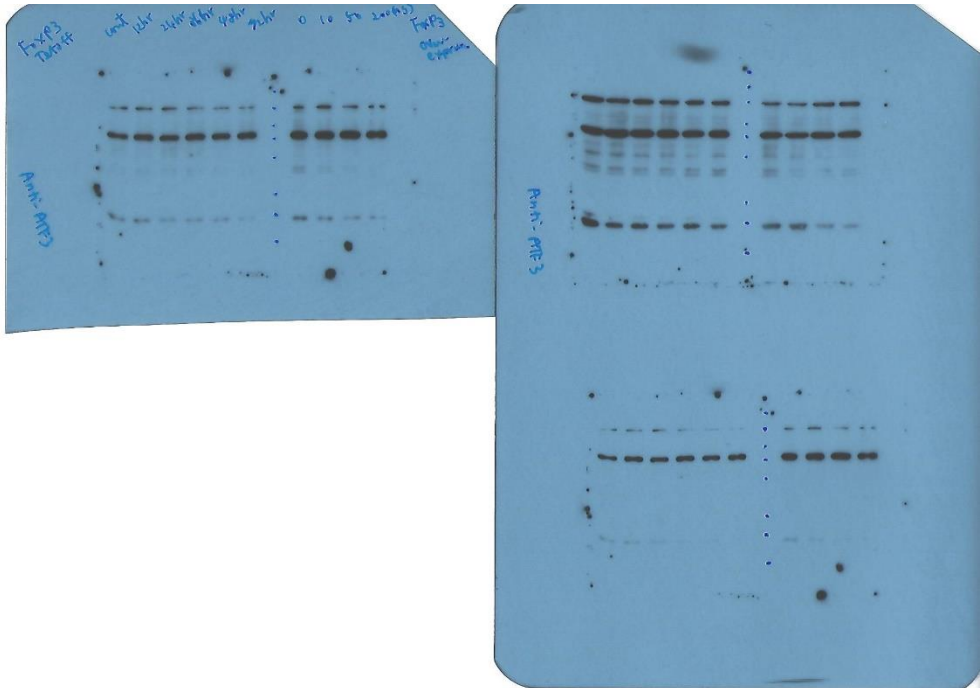

Foxp3 siRNA data

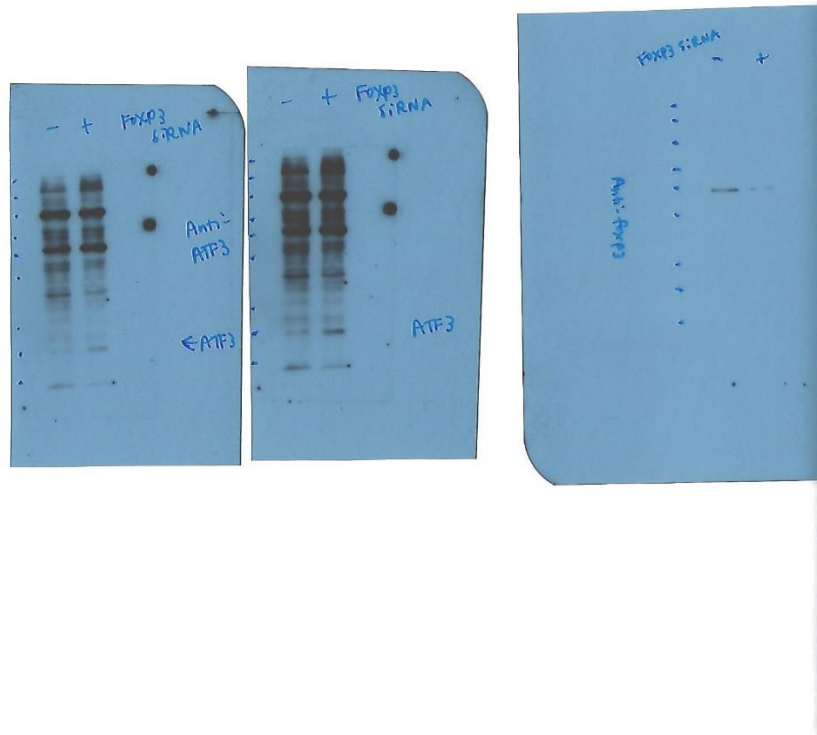

WB reporter data (Figures 2 and 4)

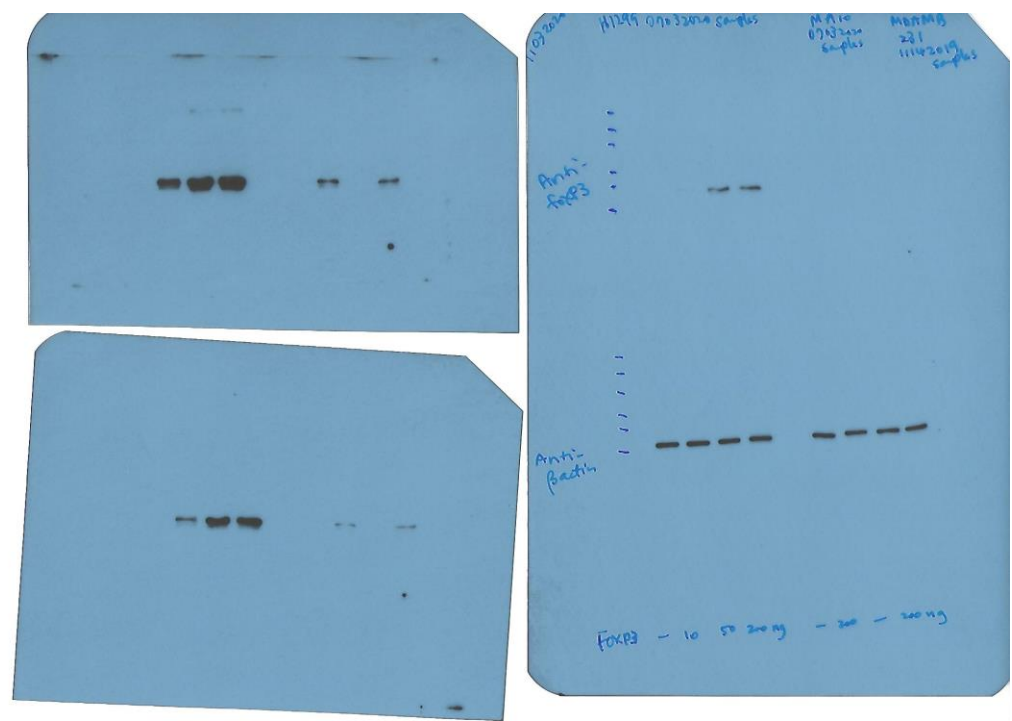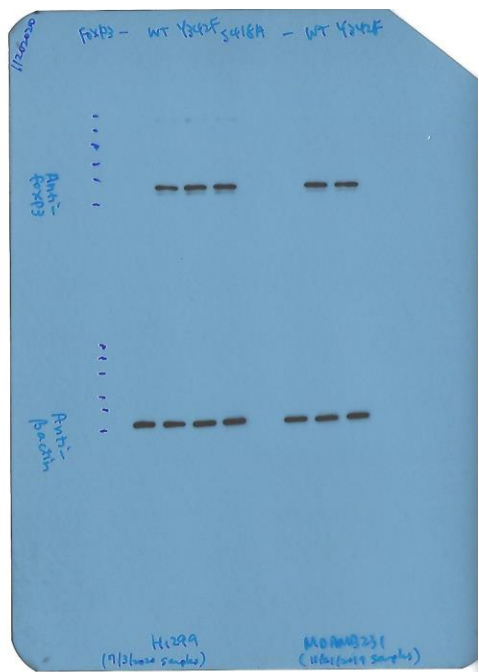

Supplement: Supplementary file 1 [file ijms-22-11400-s001.zip › ijms-1373207-supplementary.pdf]
